# A National Communications Campaign to decrease childhood stunting in Tanzania: an analysis of the factors associated with exposure

**DOI:** 10.1186/s12889-022-12930-6

**Published:** 2022-03-18

**Authors:** Ryan Moffat, Alexis Sayer, Kiersten DeCook, Alise Cornia, Mary Linehan, Scott Torres, Generose Mulokozi, Benjamin Crookston, Cougar Hall, Josh West

**Affiliations:** 1 Department of Public Health, Brigham Young University, Provo, UT USA; 2 College of Dental Medicine, Roseman University, South Jordan, UT USA; 3 IMA World Health, Washington, DC USA

## Abstract

**Background:**

Childhood stunting is a major problem in Tanzania, affecting an estimated 2.7 million children under 5 years of age. The purpose of this study was to examine the factors associated with exposure to mass media (radio and television) and IPC (interpersonal communication) components integrated in a national communications campaign aiming to decrease stunting in Tanzania.

**Methods:**

A cross-sectional survey was conducted among 3082 men and 4996 women dyads after the campaign. The average age of men was 34.7 years (SD = 8.9) and 28.1 years (SD = 6.9) for women. Several factors affecting exposure to the campaign were studied. Comparisons were made between radio, TV, and IPC exposure.

**Results:**

Mothers who reported i) higher wealth, ii) being the primary decision-makers in the home, iii) receiving support from their husbands, iv) frequent access to radio and TV and, v) ownership of a cell phone, were more likely to report exposure to the mass media component of the communications campaign. Contrarily, the same factors were not predictors of exposure to the IPC component. Fathers who reported: i) higher wealth and education, ii) ownership of a cell phone, iii) recently listened to the radio, iv) that the mother made the decisions in the home and v) helping at home, were more likely to be exposed to the mass media component.

**Conclusion:**

Significant factors affecting exposure to the communications campaign were varied but not consistent between mass media and IPC. Because of the high frequency of exposure to the campaign overall, both media and IPC components are important in a large-scale, health-related communications campaign.

## Introduction

Childhood stunting is defined as a height-to-age ratio that falls below − 2 standard deviations from the median of the World Health Organization (WHO) child growth standards [1]. Stunting results in impaired growth in a child due to repeated infection, poor nutrition, and lack of psychosocial stimulation and has been shown to be irreversible after 5 years of age [2, 3]. Stunting is a significant predictor of child development, specially during the first 1000 days of life (from conception to the child’s second birthday). About one-third of children in low-and-middle income countries, under 2 years of age are likely to be stunted [4]. Stunting is associated with lower socioeconomic status, higher number of enteropathogens in non-diarrheal stools, lower percentage of energy from protein, shorter maternal height, and lower weight-for-age [5]. Decreased chance of childhood survival, reduced cognitive functioning, hindered brain development, and increased morbidity and mortality can all result from stunting [6, 7]. Early-life stunting has been shown to affect later-life outcomes [8]. Long-term implications of stunting include decreased wages in adulthood and poverty among families due to shorter height for adults and lower school achievement [7].

In 2015, the rate of stunting in Tanzania was estimated to be 35% among children under 5 years of age [9]. Risk factors of stunting for children in Tanzania include lack of adequate nutrition, and poor water quality, sanitation, and hygiene practices (WASH) [9]. In 2015, malnutrition in Tanzania affected an estimated 600,000 children under-five years old, with 2.7 million Tanzanian children being stunted [10]. Many Tanzanian children do not have access to diets that meet their daily nutritional needs due to low meal frequency, smaller portion sizes, low nutrition content, and limited food variety [11]. While exclusive breastfeeding has been shown to be beneficial for nutritional needs of children in Tanzania, mothers (female caregiver hereafter known as mother) also face barriers for exclusive breastfeeding such as workload within the home [12, 13].

Communication distribution efforts are often implemented to create awareness of issues in a community, state or region [14]. The use of media, for example, has been proven to be an effective strategy to publicize health information, to shape health norms, and to influence a mother’s nutrition and feeding practices [15]. To this end, a large communications campaign took place in Tanzania and incorporated the constructs of Social Cognitive Theory (SCT). The SCT states that environmental influences, cognitive influences, and self-efficacy have an impact upon behavior change [16]. Other communications campaigns, such as a similar national campaign to reduce stunting in Indonesia, have utilized the SCT by having actors that are culturally or ethnically similar to the audience model desired behaviors [17]. Such modeling can increase the self-efficacy of program viewers to change behaviors that adversely affect childhood nutrition status [14]. Incorporation of the constructs of the SCT has been shown to be effective in informing program development related to parental feeding practices [18]. Like these efforts, SCT was used in this campaign to improve self-efficacy through modeling target behaviors, influence healthy social norms by depicting male involvement scenarios as normative, and to convey functional health knowledge through the provision of information and facts.

The communications components are the focus of the current study. Whereas the impact of communications campaigns on health behaviors in rural, developing settings has been studied, the factors associated with the exposure to communications campaigns in rural Tanzania are largely unknown [19]. The purpose of this study was to examine the factors associated with exposure to mass media components (radio and television) and IPC component the Addressing Stunting in Tanzania Early (ASTUTE) campaign.

## Methods

Addressing Stunting in Tanzania Early (ASTUTE) was a regional campaign that took place in the northern Lake Zone regions of Tanzania over a 4.5-year period (December 2015–May 2020) in order to inform the behavior of 3 million mothers, caregivers, and decision-makers. The purpose of ASTUTE was: i) to strengthen coordination with government nutrition sectors, and ii) to strengthen mass media and interpersonal communications (IPC) components focused on improving children’s nutrition and development indicators before a child reaches the age of 2, in order to decrease stunting. Targeted to this geographical area because of high rates of stunting, this behavior-change communications campaign included radio and TV campaigns, support groups, mobile outreach, home visits, home food production, and the training of community health workers to lead interventions about water, sanitation and hygiene. At a more local level (e.g., district, ward, and village) the IPC campaign consisted of home visits and support groups for mothers. IMA World Health (Interchurch Medical Assistance), in close collaboration with the Tanzanian Local Government Authorities and Ministry of Health, implemented this program with funding from UKaid and the Department for International Development (DFID). Key themes of the program included promotion of improved maternal health and nutrition during pregnancy; exclusive breastfeeding for children 0–6 months; complementary feeding for children 6–24 months; early child development; water, sanitation, and hygiene practices; and diarrhea treatment [20].

In this campaign, theory-based, 60-s radio spots were broadcast 10 times per day for a total of 70,000 times and each ended with a consistent tagline, a laughing baby. The TV spots were also 60 s and were aired on 3 different stations before and during the evening news on national and regional stations for a total of 1198 times. The IPC component of the intervention was implemented by community health workers (CHW) during 30-min in-home visits. The nearly 6000 CHWs counseled mothers an average of 3.6 times in-home and referred children with mother-reported faltering growth to the health facilities for treatment, educated and supported mothers to initiate timely and exclusive breastfeeding for 6 months, and empowered families to feed their children appropriately and adequately.

### Study design

The current study utilized a cross-sectional survey of 3084 men and 4996 women dyads with children aged 0–23 months. Individuals were surveyed after the communications campaign. Survey questions were directed to the mother of the youngest child in the household, as well as the father (male caregiver hereafter known as father), if available. Informed consent was received from all study participants—written if the mother was literate and by thumb print if not. The National Institute for Medical Research in Tanzania and relevant local government authorities authorized the research (TZ: NIMR/HQ/R.8a/Vol.IX/2344). Understanding that their participation was voluntary, participants could elect to terminate the survey at any time. The purpose of the questionnaire was to measure participants’ exposure to the communications campaign and other variables, including demographic and psychosocial variables related to exposure. The questionnaire was written in English, then translated to Swahili by the SMI/Ipsos team. The questionnaire was piloted and edited.

### Data collection and sampling

Data were collected by a field team consisting of 10 supervisors and 50 enumerators. All field team members participated in a two-week training in January 2020. The goal of the field team members was to recruit 5000 households. A stratified, multi-stage random sample design was used to select the survey participants. In the first stage, 243 villages were selected and participants within each village were randomly sampled. A total of 4996 mothers and 3084 fathers were surveyed from January to February 2020. Data were collected digitally with the use of smartphones and PDAs (personal digital assistants). Data quality was checked by 11 controllers, and a new interview was conducted if the quality of a previously completed interview could not be validated. Raw data were checked for outliers and invalid answers.

### Measurement

The study survey collected household and demographic information from mothers and fathers, when available. This included information about age, level of education, living in a rural vs. urban setting, and information about wealth. An adapted wealth index variable was created following procedures by Briones [21]. This variable includes access to safe water and sanitation as well as ownership of a bicycle, motorcycle, automobile, boat, and animal-drawn cart. The score is the average of the goods and services scores with a value ranging between 0 and 1; wealth increases as the value approaches 1. Frequency of viewing TV and listening to radio were both gathered. With both variables, respondents reported how often they view or listen to TV and radio and the possible response categories for both variables included never, more than a month ago, in the last month, in the last 7 days, yesterday, and today. Both of these variables were treated as continuous in the analyses.

Variables were created to denote exposure to the ASTUTE intervention and included radio, TV, and IPC exposure. A single binary definition was created for each of the campaign component variables. Exposure to radio was defined as “reported ‘yes’ to hearing the example spot/ a spot that ends with a ‘laughing’ baby sound” or “reported hearing messages on the radio that give advice about maternal/child health/child development”. Exposure to TV was defined as “reported ‘yes’ to seeing the example image frame on TV” or “reported seeing messages on the TV that give advice about maternal/ child health/ child development”. Any media campaign exposure was defined as “exposure to radio or television components of the campaign”. The IPC component of the intervention was only intended for mothers and only mothers responded to questions about exposure. Respondents were presented with a variety of health and nutrition topics and asked whether or not a CHW had visited their home to discuss these topics (yes/no).

### Statistical methods and analysis

The raw dataset containing the results from the survey was cleaned and analyzed using STATA version 16. Any missing data for key exposure variables were excluded from analyses. A significance level of *p* < .05 was used to assess the strength of the associations between independent variables and media and IPC exposure using multivariate logistic regression analyses.

## Results

Table 1 presents demographic information of study participants. The majority of mothers reported being 20–29 years of age (56.9%). Most mothers and fathers reported completing primary school, 56.5 and 64.2%, respectively. Approximately 86% of respondents lived in a rural setting.

**Table 1:** Demographics, *n* = 8078

Variable	Category	Number	Percent
Mother’s Age			
	< 20	222	4.44%
	20–24	1495	29.92%
	25–29	1366	27.34%
	30–34	953	19.08%
	35–39	534	10.69%
	40+	426	8.53%
Mother’s Education			
	Less than primary school	1481	29.65%
	Completed primary school	2840	56.86%
	Some secondary school or more	674	13.49%
Father’s Education			
	Less than primary school	529	17.16%
	Completed primary school	1981	64.28%
	Some secondary school or more	572	18.56%
Setting			
	Urban	700	14.01%
	Rural	4296	85.99%
Mean Wealth		0.27 (mean)	0.17 (std. dev.)

Table 2 contains information about psychosocial characteristics of the study sample. More than 49% of mothers and 78% of fathers reported owning a mobile phone. Nearly as many mothers reported that their husband frequently helped at home during pregnancy as reported that their husband did not frequently help at home (53% vs. 47%). Less than half of mothers received money from their own employment (43.5%) versus receiving money from some other source. At the time of the survey, most mothers reported that it had been more than a month since they had watched TV (44.9%), and over 30% of respondents reported that it had been more than a month since they had listened to the radio. The majority of fathers reported that it had been a week or less since they had last watched TV (57%) or listened to the radio (58%).

**Table 2:** Mother and Father Independent Variable Frequencies

Mother Independent Variables			
*Variable*	*Category*	*Number/Mean*	*Percent/SD*
Received Antenatal Care		4088	82.47%
Number of children		3.38	2.23
Father Help at Home During Pregnancy			
	Rarely or never	1676	35.64%
	Neither frequently nor infrequently	539	11.46%
	Frequently	2487	52.89%
Get money from own employment		2175	43.53%
Decision Making at Home			
	Father Alone	1536	30.75%
	Mother alone makes decisions	1821	36.46%
	Mother and father jointly make decisions	1502	30.07%
	Someone else makes the decisions	136	2.72%
Last watched TV			
	Never	342	6.93%
	More than a month ago	2212	44.85%
	In the last month	715	14.50%
	In the last 7 days	779	15.79%
	Yesterday	567	11.50%
	Today	317	6.43%
Last Listened to radio			
	Never	101	2.05%
	More than a month ago	1521	30.85%
	In the last month	675	13.69%
	In the last 7 days	897	18.19%
	Yesterday	880	17.85%
	Today	856	17.36%
Owns a cellphone		2482	49.68%
**Father Independent Variables**			
*Variable*	*Category*	*Number*	*Percent*
Get money from own employment		3028	98.25%
Last listened to radio			
	Never	835	27.18%
	More than a month ago	27	0.88%
	In the last month	437	14.23%
	In the last 7 days	358	11.65%
	Yesterday	708	23.05%
	Today	707	23.01%
Last watched TV			
	Never	65	2.12%
	More than a month ago	752	24.52%
	In the last month	510	16.63%
	In the last 7 days	878	28.63%
	Yesterday	661	21.55%
	Today	201	6.55%
Owns a cellphone		2417	78.42%

Table 3 shows the frequency of exposure to the ASTUTE intervention (radio, television, IPC) for mothers and fathers. Most of the mothers reported exposure to media-based interventions (60.2%) with more exposure to radio (56.5%) than television (23.5%), while just over 19% were exposed to IPC components. Similarly, fathers reported more exposure to radio messaging (65.9%) than to television messaging (38.3%).

**Table 3:** Mother and Father Dependent Variable Frequencies

**Mother Dependent Variables**		
Variable	Number	Percent
Exposed to SBCC radio message	2822	56.49%
Exposed to SBCC TV message	1175	23.52%
Exposed to either SBCC radio or TV message	3008	60.21%
Exposed to IPC visit or support group	980	19.62%
**Father Dependent Variables**		
Variable	Number	Percent
Exposed to SBCC radio message	2014	65.92%
Exposed to SBCC TV message	1154	38.28%
Exposed to either SBCC radio or TV message	2164	70.63%

Table 4 shows the factors associated with reported intervention exposure for mothers.

**Table 4:** Factors associated with intervention exposure for mothers, *n* = 4996

Variable		Mother Radio Exposure			Mother TV Exposure			Mother IPC Exposure		
		OR	CI	*P* value	OR	CI	*P* value	OR	CI	*P* value
Received Antenatal Care		1.686	1.413–2.011	<.0 01	1.429	1.129–1.808	.003	1.969	1.551–2.499	<.001
Husband Helps at Home		1.181	1.098–1.270	<.001	1.114	1.019–1.218	.017	1.170	1.076–1.272	<.001
Gets money from own employment		1.163	1.015–1.333	.030	0.951	0.806–1.121	.547	1.264	1.086–1.471	.003
Number of children		0.973	0.927–1.021	.259	0.914	0.860–0.971	.003	1.107	1.049–1.168	<.001
Decision Making at Home	Father alone (referent)	–	–	–	–	–	–	–	–	–
	Mother alone makes decisions	1.385	1.173–1.636	<.001	1.181	0.969–1.440	.099	1.030	0.851–1.247	.758
	Mother and father jointly make decisions	1.343	1.139–1.585	.001	1.072	0.876–1.311	.502	1.336	1.111–1.606	.002
	Someone else makes the decisions	1.251	0.775–2.018	.359	1.553	0.914–2.640	.104	0.569	0.279–1.157	.120
TV Freq		1.045	0.989–1.104	.116	1.806	1.669–1.920	<.001	1.038	0.977–1.103	.227
Radio Freq		1.667	1.587–1.751	<.001	1.094	1.033–1.159	.002	1.008	0.955–1.063	.775
Owns a Cellphone		1.365	1.185–1.573	<.001	1.915	1.609–2.279	<.001	1.070	0.909–1.259	.416
Age		0.999	0.983–1.015	.925	1.021	1.001–1.040	.036	0.984	0.967–1.003	.093
Wealth		2.453	1.623–3.707	<.001	3.115	1.940–5.003	<.001	0.834	0.528–1.316	.435
Education	Less than primary school (referent)	–	–							
	Complete primary school	1.216	1.045–1.415	.011	1.181	0.958–1.457	.120	1.243	1.034–1.494	.020
	Some secondary school or more	2.059	1.586–2.672	<.001	2.440	1.853–3.213	<.001	1.610	1.228–2.111	<.001

### Mother radio exposure

Factors significantly related to radio exposure among mothers included receiving antenatal care (OR 1.686, CI 1.413–2.011), receiving money (OR 1.174, CI 1.020–1.346), reporting being joint decision makers (OR 1.339, CI 1.135–1.580) or a situation where the mother was the decision maker (compared to father as the sole decision maker)(OR 1.398, CI 1.184–1.651), having a husband who helped at home (OR 1.827, CI 1.099–1.272), owning a cell phone (OR 1.315, CI 1.139–1.517), and increased education level (OR 1.352, CI 1.202–1.521). A wealthy mother had 3.5 times the odds of being exposed to the program through the radio.

### Mother TV exposure

Factors significantly related to TV exposure included receiving antenatal care (OR 1.42, CI 1.121–1.798), having a husband who helped at home (OR 1.210, CI 1.024–1.225), owning a TV, radio or cell phone, wealth and education. Greater wealth gave a mother 5.5 times the odds of being exposed to the TV campaign.

### Mother IPC exposure

Factors significantly related to the IPC component exposure included receiving antenatal care (OR 1.952, CI 1.538–2.476, *p* < 0.00), receiving money (OR 1.262, CI 1.084–1.469), the number of pregnancies (OR 1.138, CI 1.056–1.227), a situation where the mother was the decision maker (compared to father as the sole decision maker)(OR 1.343, CI 1.117–1.614), and mothers with a husband who helped at home (OR 1.169, CI 1.075–1.271). Wealth, age, owning a cell phone, a situation where the mother made decisions alone and frequently watching TV or listening to the radio were not significant factors in being exposed to the program through IPC.

### Father radio exposure

For fathers, factors significantly related to radio exposure included the husband helping at home (OR 1.150, CI 1.047–1.263), mothers being the primary decision maker (OR 1.246, CI 1.003–1.549), owning a cell phone (OR 1.478, CI 1.208–1.808), a mother that received antenatal care, wealth, and education. Frequency of listening to the radio (OR .554, CI 0.517–0.594) was significant, but negatively associated with father radio exposure to the campaign as seen in Table 5. Wealthy fathers had 3.2 times the odds of being exposed to the program through the radio.

**Table 5:** Factors associated with intervention exposure for fathers, *n* = 3082

Variable		Father Radio Exposure			Father TV Exposure		
		OR	CI	*P*-value	OR	CI	*P*-value
Received Antenatal Care		1.589	1.289–1.959	<.001	1.757	1.376–2.243	<.001
Helps wife at Home		1.183	1.084–1.292	<.001	1.198	1.090–1.317	<.001
Gets money from own employment		1.764	0.983–3.165	.057	0.986	0.528–1.842	.965
Decision Making at Home	Father Alone (referent)	–	–	–	–	–	–
	Mother alone makes decisions	1.310	1.068–1.608	.010	1.647	1.335–2.033	<.001
	Mother and father jointly make decisions	1.118	0.929–1.346	.237	1.222	1.002–1.489	.047
	Someone else makes the decisions	0.769	0.302–1.956	.581	1.622	0.597–4.408	.343
Father Cellphone		1.681	1.394–1.027	<.001	1.563	1.251–1.953	<.001
Father Radio Freq		0.889	0.857–0.922	<.001	0.926	0.894–0.959	<.001
Father TV Freq		1.228	1.150–1.312	<.001	1.929	1.791–2.079	<.001
Wealth		2.902	1.769–4.762	<.001	2.289	1.382–3.792	.001
Father Education	Less than primary school (referent)	–	–	–	–	–	–
	Complete primary school	1.073	0.869–1.325	.514	0.953	0.749–1.213	.701
	Some secondary school or more	2.156	1.595–2.914	<.001	1.861	1.389–2.494	<.001

### Father TV exposure

Factors significantly related to TV exposure included a mother that received antenatal care (OR 1.667, CI 1.304–2.123), a father that helped in the home (OR 1.194, CI 1.085–1.313), a situation where the mother makes the decisions at home (as opposed to joint decision making) (OR 1.639, 1.327–2.03), fathers and mothers jointly make the decisions at home (OR 1.240, CI 1.016–1.514), owning a cell phone (OR 1.449. CI 1.156–1.816), watching TV frequently (OR 1.840, CI 1.704–1.1.986), listening to the radio frequently (OR 0.954, CI 0.918–0.991) and, a higher level of education (OR 1.184, CI 1.101–1.274). Similarly to mother TV exposure, fathers who were wealthy had much higher odds of being exposed to the TV portion of the campaign (OR 2.90, CI 1.768–4.757).

## Discussion

The purpose of this study was to identify the factors associated with exposure to the mass media (radio or TV) components, as well as the IPC component of the ASTUTE intervention. Wealthier mothers, who were the primary decision makers in the home, had support from their husband, had frequent access to radio and TV and owned a cell phone were more likely to report exposure to the radio and TV components of the ASTUTE campaign. Contrarily, some of those same factors, higher wealth, frequent access to radio and TV, owning a cell phone, and mother being the primary decision-maker in the home were not predictors of exposure to the IPC component. Instead, mothers who had support of their husbands and made joint decisions in the home, as well as received antenatal care and money from their own employment were more likely to be exposed to the IPC component.

Fathers were included in the IPC component of the intervention whenever they were present at home during the visit, but data were not gathered on father participation. Fathers who reported higher wealth and education, owned a cell phone, recently listened to the radio, reported that the mother made the decisions in the home, and who helped at home were more likely to be exposed to the mass media components of the communications campaign.

The finding that the factors for mother exposure to the mass media and IPC components were different is unexpected but could be very useful in planning future interventions. According to previous studies, mass media tends to influence societal norms, while IPC influences personal skills [22]. Mass media campaigns have been found to increase the frequency of interpersonal discussion about a particular health issue within an individual’s social network. Over time, health behaviors promoted by the campaigns become the standard within that network [23]. Combining mass media with IPC is more likely to promote a change in behaviors [22, 24].. When used together with mass media, IPC acts as a mediator of media effects and is a functional agent for behavior change [24–27].. An individual may acquire knowledge through repeated exposure to radio or television in rather impersonal tones, but a more profound impact on beliefs and attitudes occurs when an interpersonal experience, in a comfortable setting, from an individual with technical skills, reinforces the media message [28]. The results of this study suggest that exposure to varying instruments of communication, including radio and TV campaigns, support groups, mobile outreach, home visits, homestead food production, and the training of community health workers are a successful strategy for disseminating knowledge and influencing behaviors. Although the possible interactions between the mass media and IPC components was beyond the scope of the current study, future research could identify ways to improve the overall impact of interventions prioritizing both platforms.

A high number of study participants reported exposure to the ASTUTE campaign. Other studies of large-scale health-related campaigns in developing settings have reported similar encouraging results [29]. The purpose of these campaigns is to expose large numbers of the target population to knowledge that will bring about behavior change [23].. Well-implemented media campaigns have been shown to change social norms and behaviors in positive ways [23, 30, 31]. Compared to the media component, a much smaller proportion of respondents reported exposure to IPC (60.2% vs. 19.6%). This is likely because the level of effort required to participate in IPC vs. mass media was higher, including home visits and attending classes. The results of this study suggest that respondents reporting exposure to IPC may not have easy access to mass media, but it may also be that they prefer face-to-face communication. Regardless, this study suggests that both mass media and IPC are important instruments in communication. Future successful interventions may focus on strengthening the IPC component in order to augment media effectiveness and to provide for individuals that prefer not to use mass media technologies. Incentives for participation in IPC are suggested [26]. Future interventions in rural settings may find success by continued support of more traditional approaches, such as face-to-face interaction, since ownership of devices and access to mass media was not a factor in IPC exposure.

A specific aim of ASTUTE was to establish subjective and social norms supportive of practices and attitudes that are likely to decrease stunting, persuading Tanzanian mothers and their social support networks to embrace these activities. According to the Theory of Planned Behavior (TPB), social networks can be a powerful predictor of behavioral intent. Both perceived subjective and social norms (e.g., participation in antenatal anti-stunting care and that these activities are supported by society) are powerful predictors of one’s intentions to engage in a particular health behavior [32]. By focusing on the knowledge and self-efficacy of the individual at the IPC level, ASTUTE was then able to use media to influence social and subjective norms within the community regarding these activities.

The ASTUTE communications components have been shown to be successful and powerful in the region studied [33]. Future campaigns in other regions designed to promote good practices to guard against stunting should include extensive formative research to ensure messaging platforms that are culturally specific and tailored to the target audience. Such interventions should seek to increase knowledge in caregivers that will improve attitudes and beliefs supportive of anti-stunting activities. Doing so will lead to the establishment of subjective and social norms and increase a caregiver’s feelings of perceived behavioral control or self-efficacy. Although mass media has been shown to be effective, where possible, interventions should also include interpersonal communication as these efforts have the ability to reach individuals who may be missed by mass media and are similarly effective in changing social and subjective norms. Additional research is needed to better understand how these communication campaigns can be fine-tuned and linked to other stunting reduction interventions to overcome barriers for those caregivers who find themselves unable or unwilling to engage in anti-stunting activities.

### Limitations

The results of this study must be considered in the context of its limitations. Data were gathered from only five regions in the Lake Zone of Tanzania and may not be representative of the entire Tanzanian population. Self-reporting errors may be present in relation to the cross-sectional surveys used; respondents may have been hesitant to respond accurately due to social stigma and pressure. Although participants were randomly stratified, the three cross-sectional surveys were not taken uniformly by the same individuals or the same number of individuals.

## Conclusion

Factors associated with exposure to the communications campaign were multiple and varied but not consistent between mass media and IPC. However, based upon the high frequency of individuals reporting exposure to the campaign, both media and IPC components are important in a large-scale communications campaign. Noting the differences between the two components, a focus for future practice may be to continue with mass media efforts on radio and television while strengthening participation in IPC for individuals who do not use communication technologies.

## Data Availability

The datasets generated and/or analyzed during the current study are not publicly available due to proprietary ownership by IMA World Health but are available from the corresponding author on reasonable request.
